# Related factors based on non-targeted metabolomics methods in minor ischaemic stroke

**DOI:** 10.3389/fendo.2022.952918

**Published:** 2022-09-14

**Authors:** Chen Chen, Xiaoyuan Qiao, Jianyong Guo, Ting Yang, Min Wang, Yipeng Ma, Shuhe Zhao, Ling Ding, Hong Liu, Jintao Wang

**Affiliations:** ^1^ Shanxi Cardiovascular Hospital/Cardiovascular Hospital Affiliated to Shanxi Medical University, Taiyuan, China; ^2^ Shanxi Province Cancer Hospital/ Shanxi Hospital Affiliated to Cancer Hospital, Chinese Academy of Medical Sciences/Cancer Hospital Affiliated to Shanxi Medical University, Taiyuan, China; ^3^ Neurology Department, Taiyuan Central Hospital, Taiyuan, China; ^4^ Department of Epidemiology, Shanxi Medical University, Taiyuan, China

**Keywords:** minor ischaemic stroke, non-targeted metabolomics, stroke, trimethylamine oxidation, metabolites

## Abstract

**Objective:**

This study aimed to identify potential biomarkers associated with the occurrence of minor ischaemic stroke.

**Methods:**

Four hundred patients hospitalized with minor ischaemic stroke were enrolled in the department of neurological internal medicine in Taiyuan Central Hospital, and 210 healthy subjects examined at the Taiyuan Central Hospital Medical Center during the same period were selected. We collected information on the general demographic characteristics and fasting blood samples of the subjects. We then used untargeted metabolomic assay to measure blood glucose, blood lipids, homocysteine, and high-sensitivity C-reactive protein.

**Results:**

There were statistically significant differences between the mild ischemic stroke group and the healthy control group in smoking, hypertension, and physical activity (P< 0.05). Compared with the healthy group, the minor ischaemic stroke group showed increased lactate, pyruvate, trimetlylamine oxide levels, and lactic acid, pyruvic acid, and trimethylamine N-oxidation (TMAO) levels were statistically significant (P< 0.001). In the minor ischaemic stroke risk model, hypertension, physical activity, smoking, and elevated TMAO levels influenced the occurrence of minor stroke.

**Conclusion:**

Increased levels of lactic acid, pyruvate, and TMAO may be related to the pathophysiological changes in the minor ischaemic stroke population. High blood pressure, a lack of physical activity, smoking, and increased TMAO level were the influencing factors for the occurrence of minor ischaemic stroke. The serum metabolite TMAO may be associated with MS occurrence

## 1 Introduction

Stroke is the second major cause of death worldwide. The results of a worldwide meta-analysis in 2010 revealed that 169 and 59 million patients respectively developed stroke and died of stroke-related causes (1). The results of the third national cause-of-death survey in China showed that 2 million patients are annually diagnosed with stroke and that 80% of 1.5 million of them with stroke-related deaths are due to cerebral infarction. Cerebral infarction has become a major chronic disease that threatens the health and lives of elderly people, and it is now the leading cause of death among urban and rural residents in China, accounting for 22.45% of all-cause deaths. Stroke causes billions of direct economic losses to residents and has become a serious public health issue (2).

Minor ischaemic stroke (MIS) refers to mild neurological deficit or lacunar cerebral infarction. Clear diagnostic criteria have not yet been established for MIS, and cerebral infarction is usually defined as MIS with a National Institutes of Health Stroke Scale (NIHSS) score of 0–3 points (3, 4). MIS causes minor physical damage and few people pay attention to it. However, the prevalence of MIS in elderly populations is 12–15%.

Metabolomics refers to changes in the concentrations of endogenous small molecular metabolites with a molecular weight [(MW)<1 kDa] due to internal and external stimuli. The number of metabolites in humans ranges from 2,000–20,000. Metabolic spectrum can be generated from blood, urine, and other biological samples using magnetic resonance imaging, chromatography, mass spectrometry, and other techniques. The height or area of the peak represents the relative levels of chemical components and can be used to detect and reflect changes among metabolites during various pathological or biological metabolic stages. Untargeted metabolomics is used to test relationships among metabolites in multiple interconnected pathways. Wang et al. (5)used proton nuclear magnetic resonance (1H-NMR) imaging to study a rat stroke model and found significant changes in 27 metabolites. These potential markers include acetate, bile acids, alanine, creatine, glycine, pyruvate, glycerol, fructose, lactate, acetone, 3-hydroxyisovalerate, and 3-hydroxybutyrate. High levels of trimethylamine N-oxide (TMAO) are associated with a high incidence of atherosclerotic cardiovascular disease (ASCVD) in the context of impaired renal function (6).

We screened and followed up 400 patients with MIS over a period of 3 years using nuclear magnetic resonance (NMR) and untargeted metabolomics to generate new strategies with which to prevent and treat MIS.

## 2 Materials and methods

### 2.1 Study subjects

We selected 400 patients with clinically confirmed MIS who were hospitalized at Taiyuan Central Hospital between January 2013 and December 2015 and 210 healthy persons (controls) who were assessed at the same hospital during the same period. All of them provided written, informed consent to participate in this study, which was approved by Shanxi Medical University Science Research Ethics Committee.

#### 2.1.1 Inclusion criteria

All participants were aged ≥ 18 years and had resided in Taiyuan City for > 2 years.

The inclusion criteria for patients were as follows: acute cerebral infarction confirmed by diffusion-weighted cranial magnetic resonance imaging (MRI), degree of neurological deficits due to MIS assessed by NIHSS (7) scores as 0–3. Appendix 1 shows details of the assessment scale.

The inclusion criteria for controls comprised people who were deemed healthy after a physical examination.

#### 2.1.2 Exclusion Criteria

A history of stroke, severe infection, severe liver and kidney impairment, immune system diseases, malignant tumors, neurological diseases not associated with MIS, mental illness, and severe hematologic diseases.

### 2.2 Data collection

#### 2.2.1 General demographic characteristics

We collected the following information using a structured questionnaire: demographic (sex, age, locations of residence, educational level, marital status, occupation, family income, etc.), lifestyle habits (history of smoking, history of alcohol use, physical activity, etc.), a medical history of diabetes, hypertension, coronary atherosclerotic heart disease [coronary heart disease], and atrial fibrillation.

#### 2.2.2 MRI data

The patients with MIS were assessed using cranial NMR imaging. Acute cerebral infarction was confirmed when hyperintense signals on diffusion-weighted images corresponded to hypointense signals on analogue-to-digital conversion (ADC) maps.

#### 2.2.3 NIHSS scoring

We assessed the consciousness level, sensory neglect, and visual, motor, and cerebellar functions of the patients using the NIHSS scale based on a confirmed diagnosis of acute cerebral infarction by MRI. Scores for individual parameters were obtained using a corresponding scale. The total NIHSS score for each patient comprised the sum of each component.

### 2.3 Primary reagents and equipment

The following were purchased from the respective suppliers: a 1.5-T superconducting magnetic resonance imaging system (Siemens Healthcare GmbH, Erlangen, Germany), a −80°C freezer (Haier, Qingdao, China), a Bruker 600-MHz Avance III NMR Spectrometer with a proton frequency of 600.13-MHz (Bruker Biospin Corp., Billerica, MA, USA), a TGL-16 high-speed refrigerated centrifuge (Xiangyi Centrifuge Co. Ltd, Hunan, China), NMR heavy water (Norell, Landisville, NJ, USA), 3-(Trimethylsilyl) propionic acid sodium salt (Cambridge Isotope Laboratories Inc., Andover, MA, USA), a 7180 fully automated biochemical analyzer (Hitachi Ltd., Andover, Japan), blood glucose and lipid quantitation reagents (Leadman Biochemistry Co. Ltd., Beijing, China), a homocysteine quantitation reagent (Ortho-Clinical Diagnostics, Inc., Raritan, NJ, USA), and high-sensitivity C-reactive protein quantitation reagent (Bio-Techne, Devens, MA, USA).

### 2.4 Collection and processing of biological samples

#### 2.4.1 Collection of blood samples

Venous blood from participants within 24 h of enrolment was collected in the morning after fasting and stored with anticoagulants. Blood glucose, blood lipids, homocysteine, and high-sensitivity C-reactive protein were quantified and serum metabolomics was assessed in 5 mL samples.

#### 2.4.2 Processing of blood samples

Blood samples for quantifying blood glucose, blood lipids, homocysteine, and high-sensitivity C-reactive protein were left at room temperature for 1 h before centrifugation at 3,500 rpm for 10 min to collect serum which was immediately analyzed or stored at -20°C and analyzed within 24 h.

Blood samples for serum metabolomics analysis were immediately centrifuged at 3,500 rpm for 10 min, then serum was separated and transferred to 1.5-mL Eppendorf (EP centrifuge) tube, sealed, and stored at -80°C.

### 2.5 Quantitation of biological samples

#### 2.5.1 Quantitation of blood glucose, blood lipids, homocysteine, and high-sensitivity C-reactive protein

Blood glucose, total cholesterol, triglycerides, and high-density lipoprotein (HDL) were measured using oxidase as described by the manufacturer of the fully automated analyzer. Levels of low-density lipoprotein (LDL) were determined using a homogeneous enzymatic assay. Homocysteine and high-sensitivity C-reactive protein levels were measured using enzymatic cycling and enzyme-linked immunosorbent assays, respectively.

#### 2.5.2 Quantification of serum metabolomics

Untargeted metabolomics was quantified using NMR imaging. Serum samples were thawed in ice water, then 450 μL samples were vortex-mixed with 350-μL D_2_O for 30 seconds and centrifuged at 13,000 rpm for 10 min at 4°C and 600 μL of the supernatant in 5-mm NMR tubes were then analyzed as follows:

(1) Broad peaks of proteins and lipoproteins were attenuated using the 600-MHz NMR scanner (64 scans; spectral width, 12345.679 Hz; Fourier transformation, 0.188 Hz; pulse interval D1, of 1 second; delay, 5.0 seconds) with the Carr–Purcell–Meiboom–Gill (CPMG) pulse sequence.

(2) MestReNova v. 8.0.1 software (Mestrelab Research, Santiago de Compostella, Spain) was used to process spectral NMR maps in which the phase and baseline were manually adjusted. The chemical shift of creatinine (δ3.04) was the standard for chemical shift correction, and segments were integrated at δ0.80–8.50 chemical shift regions using δ0.01. Segments were not integrated at δ4.67–4.96 (residual water peak). The integration data were normalized and performed the multivariate statistical analysis.

(3) SIMCA-P+ software (Stedim Biotech GmbH., Göttingen, Germany) was used for multivariate and principal component analysis (PCA). PCA analysis converts a large number of correlated variables into a small set of uncorrelated variables, while preserving as much information as possible about the original variables. It can be used to replace the original large number of correlated variables, thereby simplifying the analysis process. We further applied orthogonal signal correction (OSC) followed by orthogonal partial least squares discrimination analysis (OPLS-DA) to eliminate the effects of relevant factors such as individual differences on grouping and to strengthen intergroup differences. OPLS-DA establishes the relationship model between metabolite expression and sample category by using partial least squares regression, and the sample relationship can be better established by reducing the dimensionality of the data.

### 2.6 Statistical analysis

Structural questionnaire, NIHSS scores, imaging, serum biochemistry, and metabolomics were statistically analyzed using SPSS 17.0 (SPSS Software Inc., Chicago, IL, USA). Continuous data were described as mean and standard error, and a *t* test was used for comparison between the two groups. Data from multiple groups were compared using One-way analysis of variance (ANOVA), intra-group pairwise comparisons were assessed using the least significant difference method. The quantity and ratios (%) of discrete data were analyzed using the chi-square test, and Fisher exact probability test on rows × columns. P value of<0.05 was considered statistically significant. The inclusion and exclusion criteria were 0.05 and 0.10, respectively, for multivariate analysis with logistic regression.

## 3. Result

### 3.1 General demographic characteristics and relevant factors

In this study, we enrolled 400 patients with MIS and 210 healthy controls. A comparison of general status revealed that smoking, hypertension, and physical activity significantly differed between the two groups (P< 0.05), whereas age, alcohol consumption, sex, and diabetes did not. These findings indicated that the two populations are generally similar and comparable, while smoking, hypertension, and physical activity might influence MIS (**Table 1**).

**Table 1:** Comparison of general conditions between the MIS and control groups.

Variable	Minor ischaemic stroke group (n = 400)	Control group(n = 210)	*t*/*χ* ^2^ value	P value
Age	64.40 ± 12.90	65.16 ± 11.95	0.035	0.882
Sex			0.013	0.909
Male	240 (60.0)	127 (60.5)		
Female	160 (40.0)	83 (39.5)		
Smoking			29.593	<0.001
No	272 (68.0)	185 (88.1)		
Yes	128 (32.0)	25 (11.9)		
Alcohol consumption			0.027	0.886
No	302 (75.5)	160 (76.2)		
Yes	98 (24.5)	50 (23.8)		
Hypertension			114.300	<0.001
No	116 (29.0)	156 (74.3)		
Yes	284 (71.0)	54 (25.7)		
Diabetes			0.238	0.546
No	298 (74.5)	167 (79.5)		
Yes	102 (25.5)	43 (20.5)		
Physical activity			88.824	<0.001
Less	130 (32.5)	2 (1.0)		
Yes	270 (67.5)	208 (99.0)		

### 3.2 Correlation among levels of blood glucose, blood lipids, homocysteine, and high-sensitivity C-reactive protein

The results of univariate analysis of blood glucose, blood lipids, homocysteine, and high-sensitivity C-reactive protein revealed significant differences in triglyceride, HDL, and high-sensitivity C-reactive protein levels between the two groups (P< 0.05, **Table 2**).

**Table 2:** Correlation analysis of biochemical markers in minor ischaemic stroke.

Variable	Minor ischaemic stroke group(n = 400)	Control group(n = 210)	*t* Value	P value
Blood glucose	5.80 ± 2.27	5.91 ± 2.56	−0.521	0.603
Lipoprotein a	114.01 ± 20.01	113.61 ± 15.84	0.542	0.588
Apolipoprotein A	1.29 ± 0.25	1.30 ± 0.22	−0.450	0.653
Apolipoprotein B	1.22 ± 0.29	0.82 ± 0.19	1.490	0.137
Apolipoprotein E	4.45 ± 0.74	5.28 ± 0.58	−0.011	0.991
Triglycerides	1.21 ± 0.39	1.12 ± 0.47	2.159	0.031
Total cholesterol	4.02 ± 0.61	4.00 ± 0.60	0.622	0.534
Low-density lipoprotein	2.52 ± 0.35	2.51 ± 0.34	0.188	0.851
High-density lipoprotein	1.30 ± 0.26	1.43 ± 0.28	−4.251	<0.001
High-sensitivity C-reactive protein	2.21 ± 0.94	1.84 ± 1.45	2.099	0.036
Homocysteine	8.93 ± 1.32	9.59 ± 1.74	−0.581	0.561

### 3.3 Metabolomics

**Figure 1:**
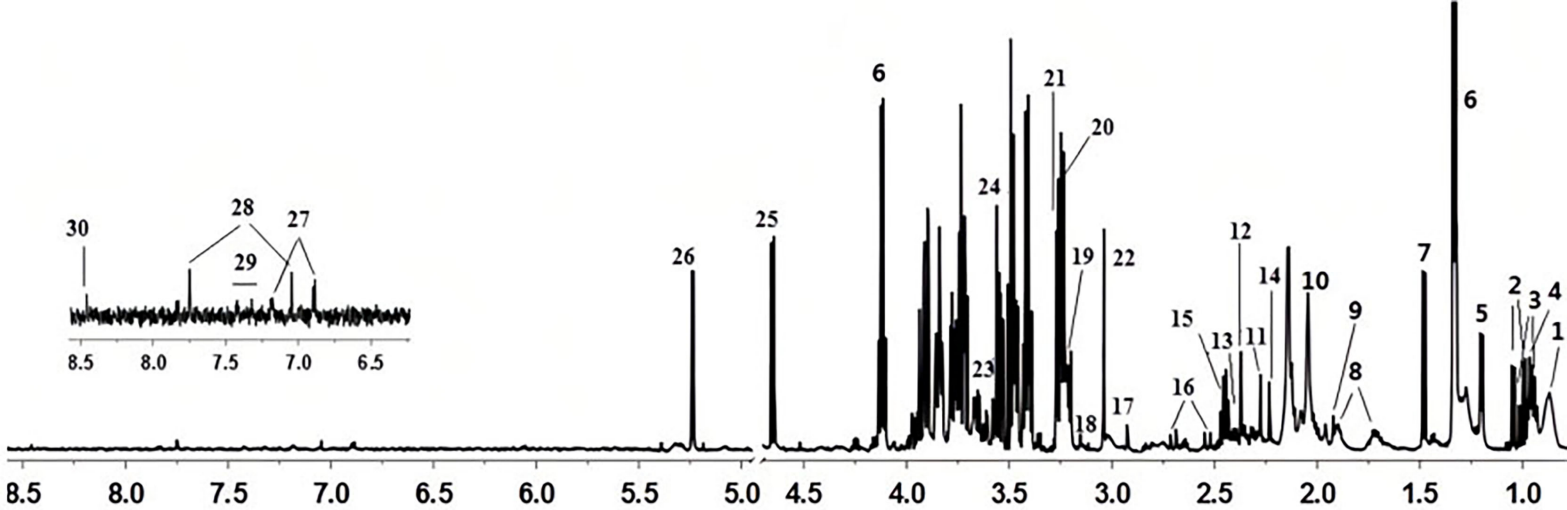
Serum nuclear magnetic resonance (NMR) maps.

Chemical shift, coupling constant, peak type, and other data in the serum NMR maps combined with the standards, analyses of the Human Metabolomics (HMDB; http://www.hmdb.ca) and Biological Magnetic Resonance Data Bank (BMRB; http://www.bmrb.wisc.edu) databases, and a comparison with the literature (8–10) identified 30 compounds (**Figure 1**, **Table 3**).

**Table 3:** Metabolites identified using metabolomics.

S/N	Metabolite	Chemical structure	Chemical shift^a^	Sample^b^
1	Lipids	CH_3_, (CH_2_)_n_, CH=CH	0.87 (m)^a^, 1.28 (m), 5.30 (m)	S^b^
2	Valine	γCH_3_, γ'CH_3_	0.99 (d), 1.05 (d)	S
3	Isoleucine	δCH_3_, γCH_3_, γ'CH_2_	0.94 (t), 1.02 (d), 1.27 (m)	S
4	Leucine	δCH_3_, δ'CH_3_	0.96 (d), 0.97 (d)	S
5	3-Hydroxybutyrate	γCH_3_, half αCH_2_	1.20 (d), 2.41 (d), 2.31 (d)	S
6	Lactic acid	αCH, βCH_3_	1.33 (d), 4.12 (q)	S
7	Alanine	βCH_3_	1.48 (d)	S
8	Lysine	βCH_2_, δCH_2_, γCH_2_	1.89 (m), 1.73 (m), 1.44 (m)	S
9	Acetate	CH_3_	1.92 (s)	S
10	NAG^c^	CH_3_	2.05 (s)	S
11	Acetoacetate	CH_3_	2.28 (s)	S
12	Pyruvate	CH_3_	2.37 (s)	S
13	Succinate	CH_2_	2.41 (s)	S
14	Glutamic acid	βCH_2_, γCH_2_	2.06 (m), 2.35 (m)	S
15	Glutamine	βCH_2_, γCH_2_	2.14 (m), 2.46 (m)	S
16	Citrate	half CH_2_, half CH_2_	2.53 (d), 2.70 (d)	S
17	Dimethylglycine	N-CH_3_, CH_2_	2.93 (s), 3.72 (s)	S
18	Choline	N (CH_3_)_3_	3.20 (s)	S
19	PC^c^	N (CH_3_)_3_	3.22 (s)	S
20	GPC^c^	N (CH_3_)_3_	3.23 (s)	S
21	TMAO^c^	CH_3_	3.27 (s)	S
22	Creatinine	CH_3_, CH_2_	3.04 (s), 3.94 (s)	S
23	Glycerol	CH_2_OH, CH_2_OH	3.66 (dd), 3.57 (dd)	S
24	Glycine	CH_2_	3.56 (s)	S
25	α-Glucose	1-CH	4.65 (d)	S
26	β-Glucose	1-CH	5.23 (d)	S
27	Tyrosine	3- or 5-CH, 2- or 6-CH	6.89 (d), 7.18 (d)	S
28	Histidine	2-CH, 4-CH	7.75 (s), 7.05 (s)	S
29	Phenylalanine	2- or 6-CH, 3- or 5-CH	7.32 (m), 7.42 (m)	S
30	Formate	CH	8.46 (s)	S

a. s, Singlet; d, doublet; t, triplet; m, multiplet; dd, doublet of doublet; b. S, serum; c. NAG, N-acetylated glycoprotein; OC, phosphatidylcholine; GPC, glycerophosphocholine; TMAO, trimethylamine oxide.

#### 3.3.1 Separation trends of serum metabolites from the MIS and control groups

**Figure 2:**
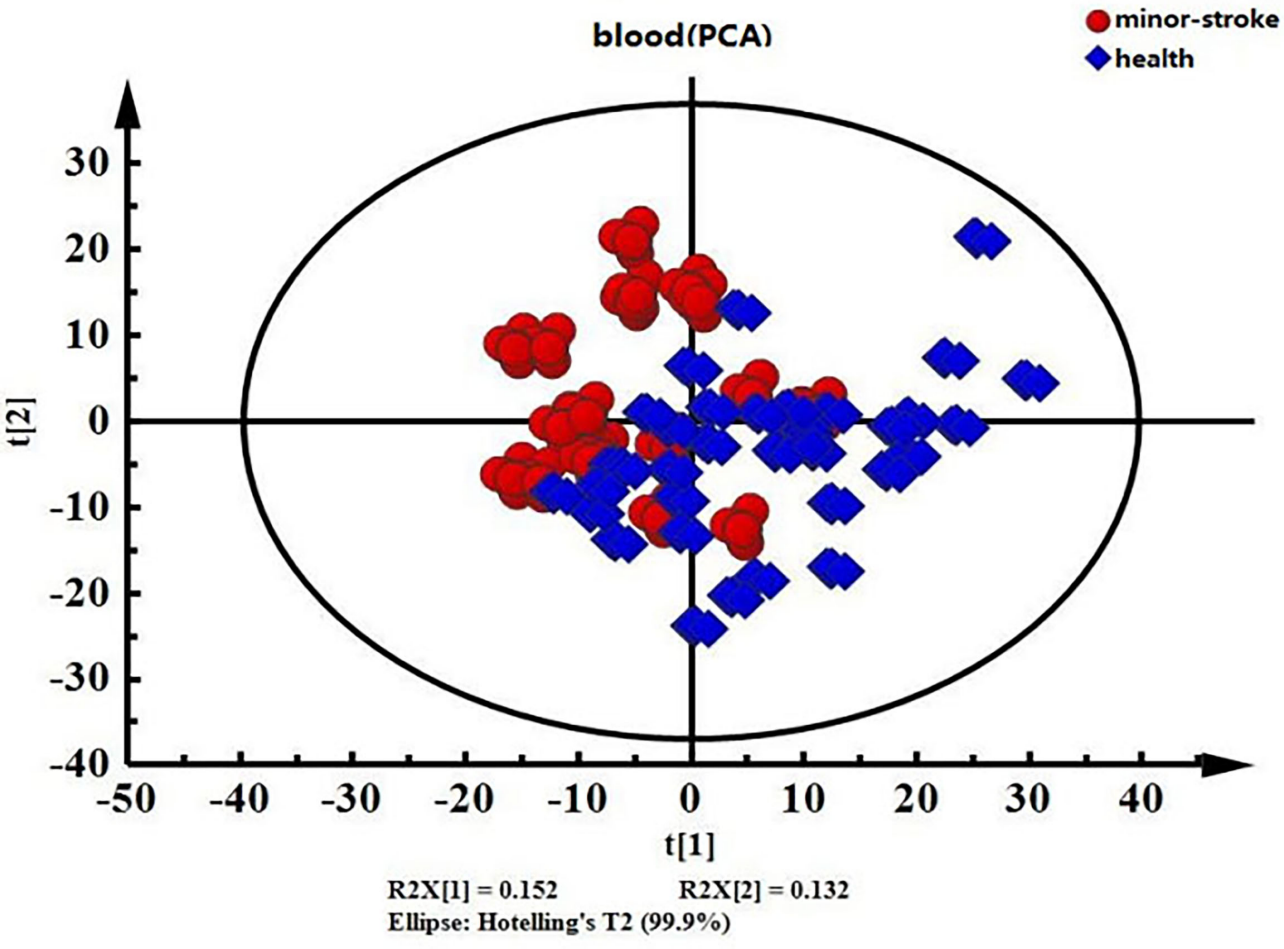
PCA score chart of serum data from the minor ischaemic stroke and control groups. Red, minor ischaemic stroke group; blue, control group.

The PCA of unsupervised pattern recognition can reflect the original state of the data and intuitively show overall differences between samples. The results of the PCA revealed a separation trend (**Figure 2**), indicating significantly different serum metabolomics between the MIS and control groups.

#### 3.3.2 Comparison of serum metabolites between MIS and control groups

**Figure 3:**
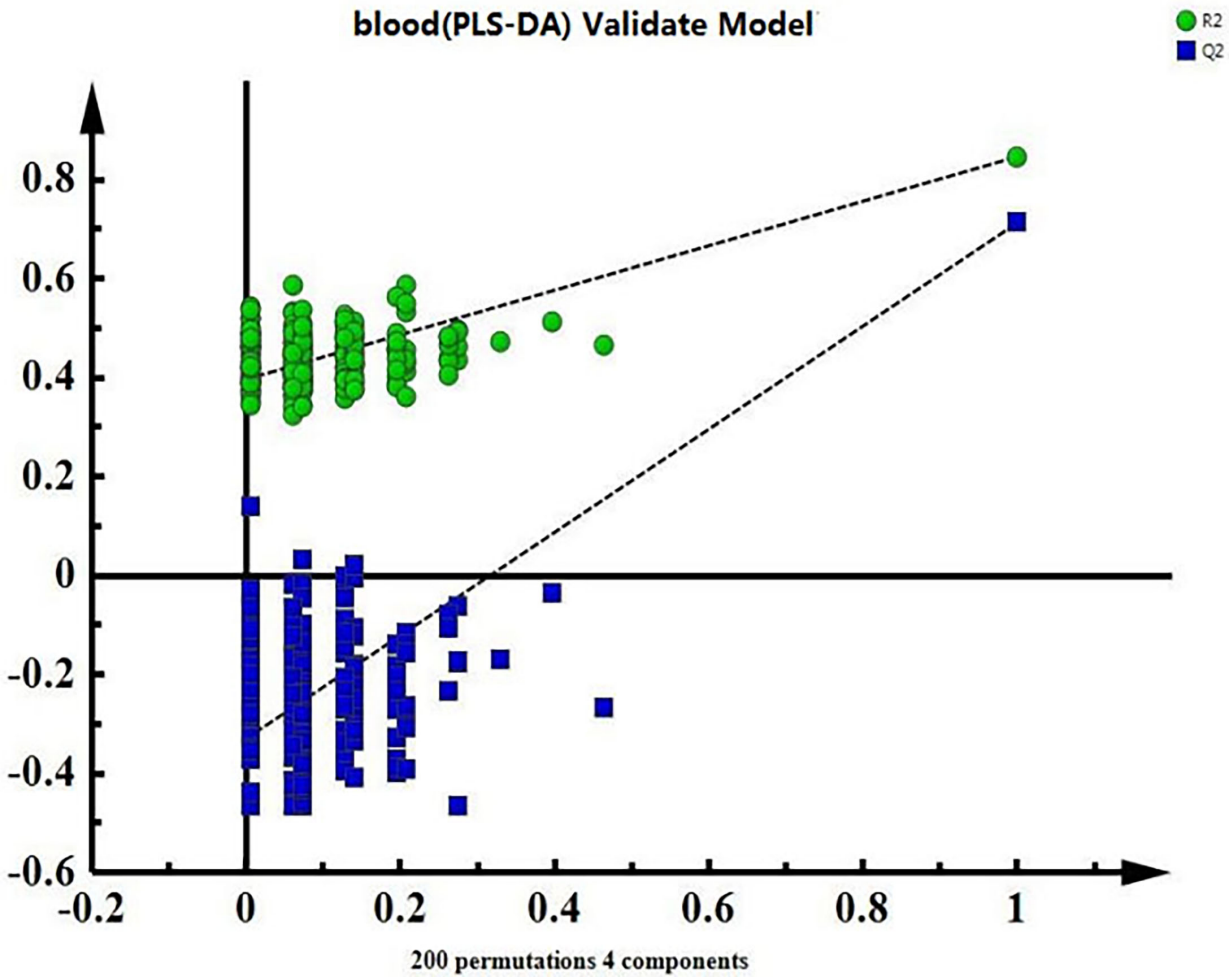
Validation graph for the serum model alignment experiment in the minor ischaemic stroke and control groups.

The OPLS-DA requires PLS-DA analysis as a basis for model validation. The alignment results showed that the model was effective and reliable (**Figure 3**) and could be used to search for differences.

**Figure 4:**
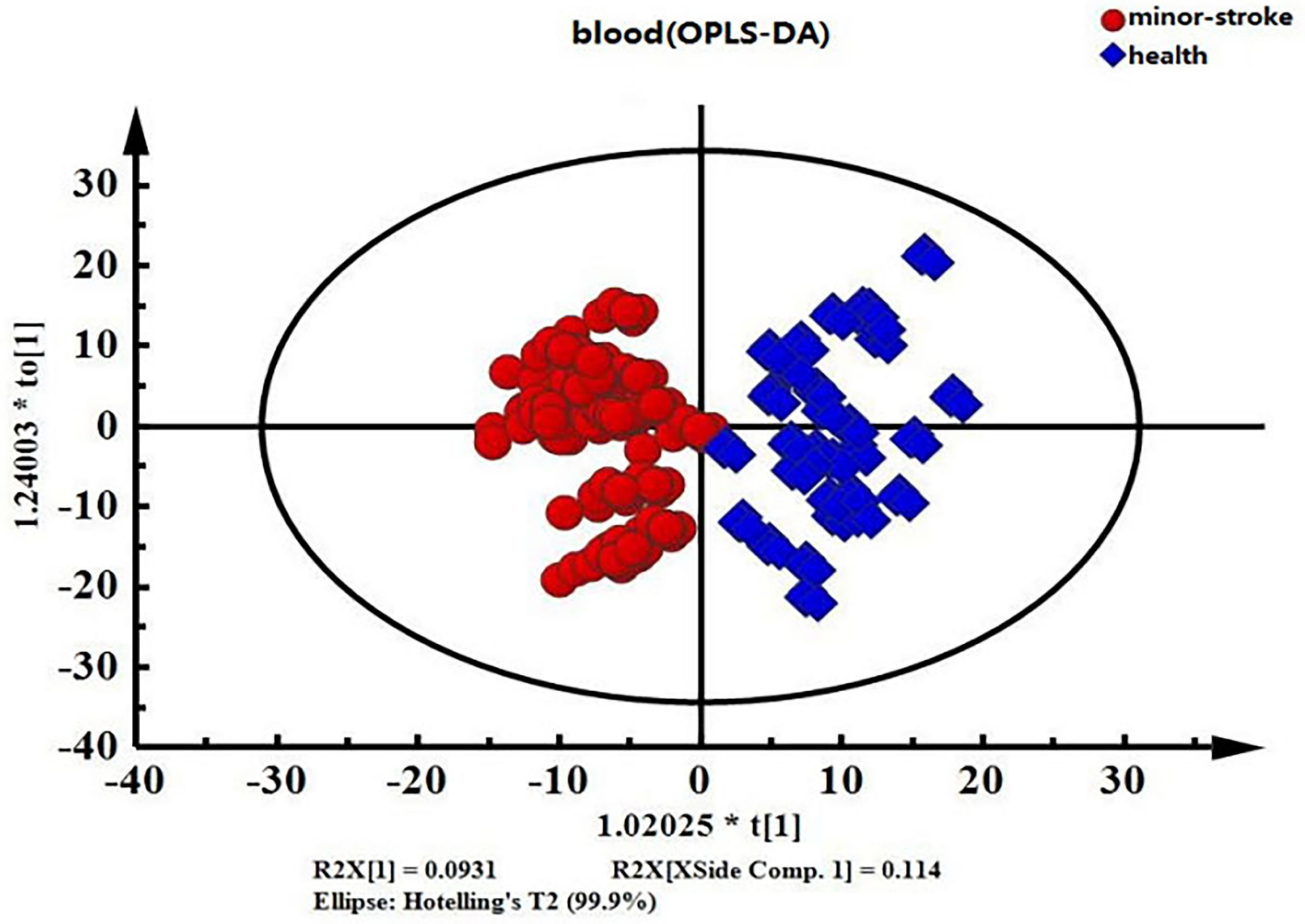
OPLS-DA score chart of serum data from the minor ischaemic stroke and control groups. Red, minor ischaemic stroke group; blue, control group.

Intra-group differences can be overlooked and random errors that are not related to the study objectives can be removed by OPLS-DA. This facilitates the accurate identification of inter-group differences and differential metabolites. Therefore, we compared differences in endogenous serum metabolites between the MIS and control groups using OPLS-DA (**Figure 4**). The OPLS-DA score chart of serum data showed significant inter-group differences.

**Figure 5:**
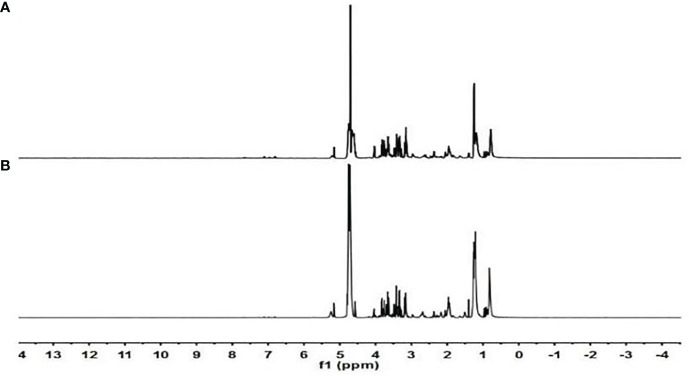
Serum NMR maps of the minor ischaemic stroke and control groups. **(A)** control group; **(B)** minor ischaemic stroke group.

**Figure 6:**
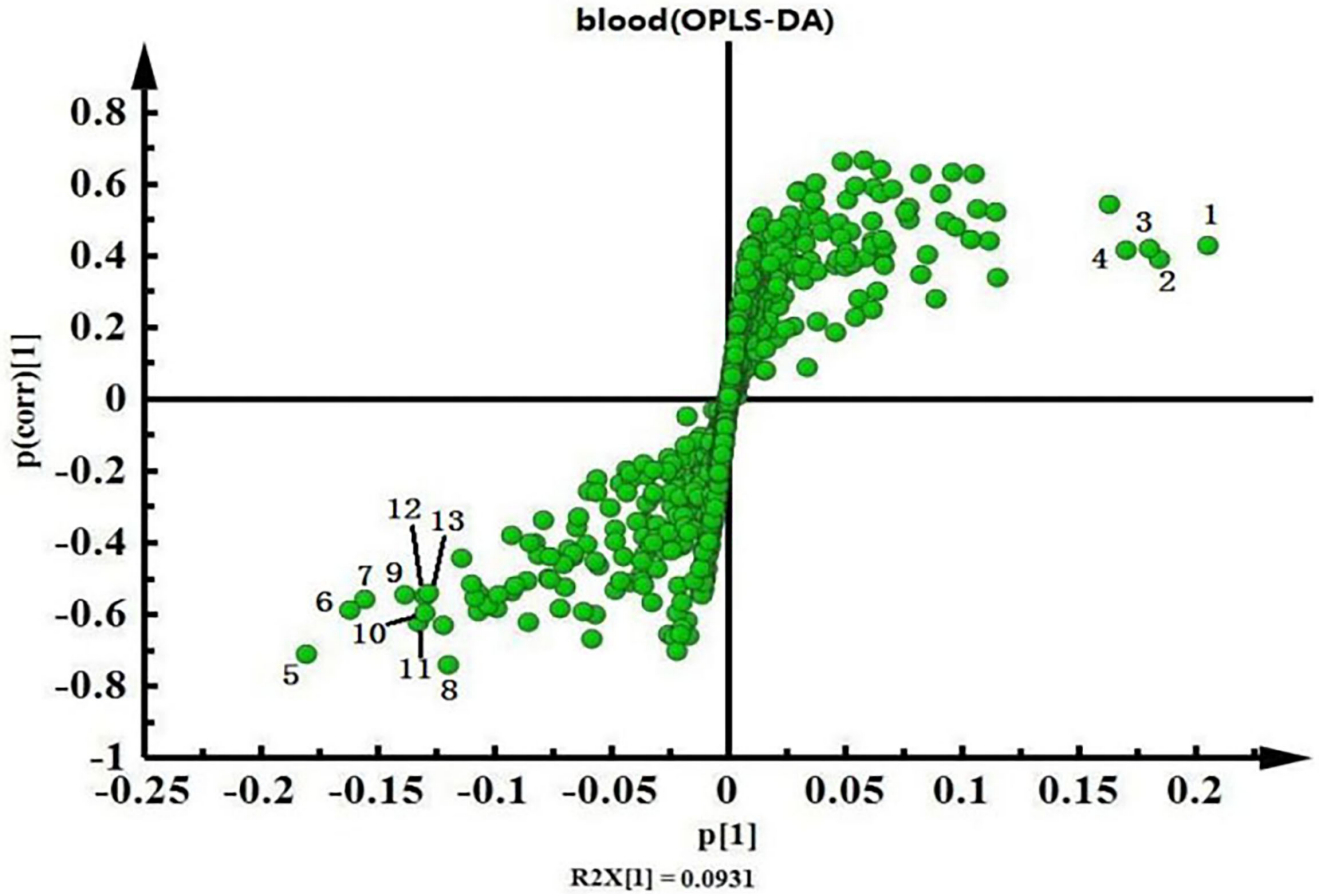
Serum data S-plot loading graphs of the minor ischaemic stroke and control groups. 1. Lipids; 2. NAG; 3. choline; 4: PC, 5: valine; 6. alanine; 7. glutamate; 8. glutamine; 9. TMAO; 10. glycine; 11. lactate; 12. β-glucose; 13. α-glucose.

**Figure 5** shows the serum NMR map of the two groups. We combined the S-plot (**Figure 6**) and VIP value (>1) and confirmed higher levels of lipids, NAG, choline, and PC in the control group than those in the MIS group. In contrast, levels of valine, alanine, glutamate, glutamine, TMAO, glycine, lactate, α-glucose, and β-glucose were higher in the MIS group than those in the control group.

Owing to the spectral properties of the NMR, we could only measure integral area of the above compounds, and the results of a univariate analysis was performed of substances in the NMR map that did not have completely overlapping signals (**Table 4**) revealed increased levels of valine, lactate, alanine, glutamate, glutamine, pyruvate, TMAO, α-glucose, and β-glucose, and decreased levels of lipids, NAG, choline, and PC in the MIS group. Among these metabolites, lactate, pyruvate, and TMAO significantly changed (P< 0.001).

**Table 4:** Analysis of differences in serum metabolites between the minor ischaemic stroke and control groups.

Metabolite	Control group	Minor ischaemic stroke group	*t* Value	P value
Valine	0.242 ± 0.011	0.254 ± 0.012	−0.842	0.201
Lactic acid	0.399 ± 0.043	0.513 ± 0.045	−5.427	<0.001
Alanine	0.317 ± 0.015	0.333 ± 0.017	−0.851	0.234
Glutamic acid	0.996 ± 0.005	0.106 ± 0.006	−0.799	0.241
Glutamine	0.129 ± 0.011	0.139 ± 0.009	−0.543	0.465
Pyruvic acid	0.071 ± 0.006	0.109 ± 0.007	−18.247	<0.001
TMAO	0.585 ± 0.045	0.814 ± 0.03	−9.926	<0.001
α-Glucose	0.571 ± 0.025	0.582 ± 0.023	−0.552	0.441
β-Glucose	0.477 ± 0.041	0.511 ± 0.012	−1.356	0.083
Lipids	1.312 ± 0.072	1.290 ± 0.049	0.957	0.188
NAG	0.207 ± 0.013	0.195 ± 0.016	0.572	0.435
Choline	0.722 ± 0.045	0.712 ± 0.031	0.447	0.607
PC	0.256 ± 0.034	0.253 ± 0.025	0.217	0.836

### 3.4 Logistic regression analysis of influencing factors of MIS

Factors that might be associated with MIS determined herein were included in the logistic regression model for multivariate analysis. Pearson's correlation analysis showed that the correlation between the covariates was not significant. Relationship between potential influencing factor and MIS, were analyzed by stepwise regression, with inclusion criterion of 0.05 and exclusion criterion of 0.10. **Table 5** showed that hypertension (odds ratio [OR] = 4.033; 95% confidence interval [CI], 2.581–6.303; *P*< 0.001), physical activity (OR = 0.026; 95% CI, 0.006–0.113, *P*< 0.001), smoking (OR = 3.104; 95% CI, 1.783–5.402; *P*< 0.001), and TMAO (OR = 1.227; 95% CI, 1.106–1.483; *P* = 0.034) affected the occurrence of MIS. Hypertension, smoking, and TMAO levels were risk factors for MIS, while physical activity was a protective factor (**Table 5**). We derived the following risk warning model for MIS:

**Table 5:** Multivariate logistic regression analysis of factors associated with the occurrence of minor ischaemic stroke.

Variable	B	SE	Wald	Sig.	Exp(B)	95% CI
Hypertension	1.395	0.228	37.468	<0.001	4.033	2.581~6.303
Physical activity	−3.638	0.742	24.007	<0.001	0.026	0.006~0.113
physical activity	-3.638	0.742	24.007	<0.001	0.026	0.006~0.113
Smoking	1.133	0.283	16.047	<0.001	3.104	1.783~5.402
Trimethylamine oxide	1.484	0.386	14.752	0.034	1.227	1.106~1.483
Constant	2.317	0.542	15.741	0.010	1.773	


logit P=2.317+1.395X1−3.638X2+1.133X3+1.484X4

where *X_1_*, *X_2_*, *X_3_*, and *X_4_* respectively represent hypertension, physical activity, smoking, TMAO.

## 4 Discussion

A comparison of 400 patients with MIS and 210 healthy controls revealed no significant differences in age, sex, and other demographic variables between them, which indicated that they were comparable. The results of serum metabolomics showed differences in valine, lactate, alanine, glutamate, glutamine, pyruvate, TMAO, α-glucose, β-glucose, lipids, NAG, choline, and PC levels between the MIS and control groups. Among these, changes in the levels of lactate, pyruvate, and TMAO were statistically significant.

We analyzed demographic characteristics, blood lipids, blood glucose, and other relevant factors to screen for factors that might affect the occurrence of MIS. We found that hypertension, smoking, physical activity, and TMAO showed statistically significant differences between the groups.

Dyslipidaemia is a risk factor for cerebro-cardiovascular diseases. Lipid deposition in vascular walls will result in vascular stenosis and reduced elasticity, which results in insufficient blood supply to target organs. Excess blood lipids deposition in arteries and tends to lead to atherosclerosis. Triglycerides and HDL levels are associated with cerebral infarction (11). Dyslipidaemia not only promotes macroangiopathy, but also clearly correlates with microangiopathy (12). Although elevated blood levels of triglycerides are positively correlated with cerebral infarction (13), they might decrease the morbidity and mortality in cerebral infarction (14). Protective functions of elevated triglycerides might be associated with the fact that triglycerides are components of the cell membrane and as such are indispensable to the maintenance of homeostasis. Triglycerides are also markers of nutritional status and are constant energy sources. Low triglyceride levels can lead to malnutrition and increase the risk of poor outcomes in cerebral infarction. Elevated triglycerides are also associated with serum albumin levels and they jointly reduce the mortality risk due to cerebral infarction (15). HDL (16) is a scavenger protein. Slightly elevated HDL levels in the controls suggested a negative correlation with the occurrence of MIS. A 21-year follow-up study (17) also showed that HDL decreases the mortality rate in stroke. Low HDL levels might increase damage due to cerebral infarction and have been associated with poorer clinical outcomes (18, 19). By contrast, increased HDL levels decrease the area of damaged brain tissues and reduce the severity of cerebral infarction to improve the prognosis of cerebral infarction (20, 21). High-sensitivity C-reactive protein is a non-specific inflammatory mediator that can activate the complement system, induce inflammation-associated endothelial injury, increase atherosclerotic plaques, and cause thrombus formation (22). High-sensitivity C-reactive protein is also correlated with the severity of brain injury. High-sensitivity C-reactive protein is an inflammatory marker with predictive value for cerebrovascular diseases (23). Inflammation is considered a risk factor for small vessel disease (SVD) (24), and it plays a significant role in SVD (25). B vitamins have anti-inflammatory properties and play a protective role in anti-neurodegenerative mechanisms (26). However, there is insufficient to draw definitive conclusions about an intermediate relationship between B vitamins and fatty acids and cognitive decline (27). Chronic cigarette smoking (28) and reduced physical activity (29) might increase the incidence of MIS because they increase blood viscosity and slow blood flow. Hypertension is a textbook example of an independent risk factor of cerebral infarction and is the leading risk factor of MIS. Approximately 90% of MIS is associated with hypertension, which can decrease the self-regulatory mechanisms of cerebral blood flow and reduce blood supply. This is because the blood flow impact of high pressure damages sclerosed vascular walls, which results in thrombus formation or rupture and leads to stroke (30, 31).

**Figure 7:**
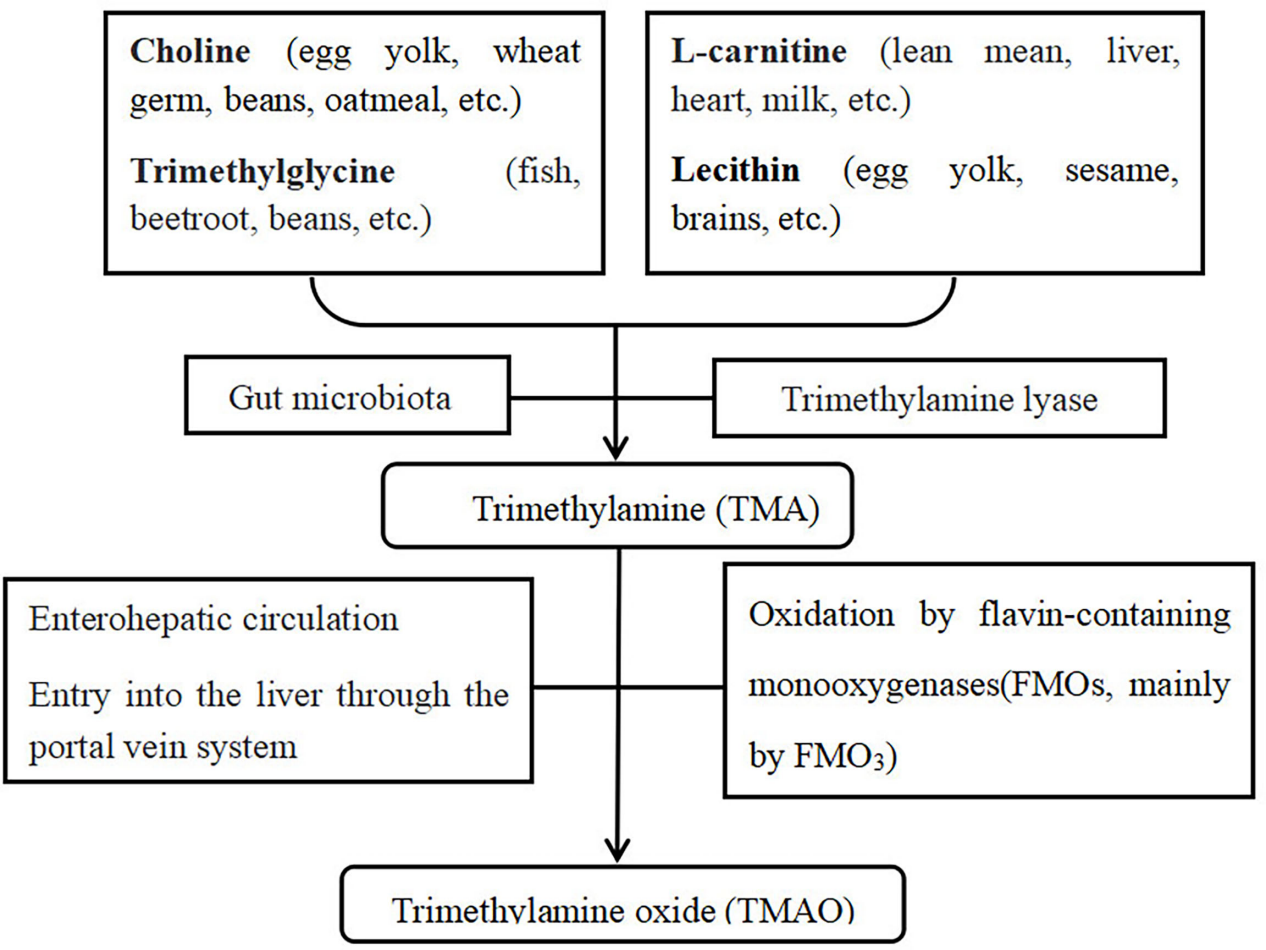
TMAO metabolic pathway.

Wang et al. found that the gut microbiota metabolism of dietary L-carnitine produces TMAO and accelerates atherosclerosis (32). Thereafter, they used a structural analogue to choline, 3-dimethyl-1-butanol (DMB) for targeted inhibition of TMA formation and TMAO synthesis to determine the effects of TMAO on atherosclerosis (33). They found that DMB inhibits the formation of endogenous macrophage and development of atherosclerotic lesions caused by choline but does not change the circulating cholesterol levels. Chen et al. (34) have found that resveratrol reduces TMAO levels in a mouse ApoE model, the occurrence of atherosclerosis, and the incidence of cerebro-cardiovascular diseases. TMAO is a new and independent risk factor that promotes atherosclerosis (35, 36). Excessive dietary intake of foods that increase TMAO levels may lead to obesity and metabolic syndrome. Chen et al. (37) have proved that consumption of a Western diet (high sugars and saturated fat) can increase circulating TMAO levels, which result in cardiac inflammation and fibrosis and cause cardiac impairment. High plasma TMAO levels strongly correlate with the development of atherosclerosis (12), and TMAO can exert metabolic control through the microbiota enterohepatic axis (38). Furthermore, TMAO can accelerate endothelial dysfunction by decreasing endothelial self-repair and increasing monocyte adhesion, which promotes the early pathological process of atherosclerosis. Dietary TMAO promotes the up-regulation of multiple macrophage scavenger receptors that are associated with atherosclerosis and the development of atherosclerotic lesions in mice (39). Wang et al. (33, 40) have suggested that trimethylamine (TMA)/TMAO participates in the development of atherosclerosis in the food-TMA-TMAO-atherosclerosis-cerebro-cardiovascular disease pathway, thereby ultimately resulting in cerebro-cardiovascular disease. The metabolic pathway of TMAO is shown in **Figure 7**.

We found that compared with healthy controls, hypertension, smoking, physical activity, and elevated TMAO are associated with the occurrence of MIS. Among these factors, hypertension and smoking are established risk factors for cerebral infarction. Appropriate physical activity is also an established protective factor that can prevent cerebral infarction. MIS is a subset of cerebral infarction, and its risk factors are evidently similar to those of cerebral infarction. We found that hypertension, reduced physical activity, and elevated TMAO levels are early warning markers of MIS. In particular, the serum metabolite TMAO may be associated with the occurrence of MS. TMAO might have functions in addition to its role as an intermediate in lipid metabolism that remains to be explored. Due to the limited sample size of this study, we will further expand the sample to conduct in-depth research on trimethylamine oxide and related substances in its upstream and downstream pathways.

## Data availability statement

The original contributions presented in the study are included in the article/supplementary material. Further inquiries can be directed to the corresponding authors.

## Ethics statement

The studies involving human participants were reviewed and approved by Shanxi Medical University Science Research Ethics Committee. The patients/participants provided their written informed consent to participate in this study.

## Author contributions

Conceptualization: CC and XQ Methodology: HL, JW Formal analysis and investigation: JG, TY, MW Writing - original draft preparation: CC and XQ Writing - review and editing: CC and JW Funding acquisition: CC and JW Resources: YM, HL Supervision: SZ, LD. All authors contributed to the article and approved the submitted version.

## Funding

This work was financially supported by the Applied Basic Research Program of Shanxi Province (201601D011098) and Four “Batches” Innovation Project of Invigorating Medical through Science and Technology of Shanxi Province (2022XM07).

## Conflict of interest

The reviewer RY declared a shared affiliation with the authors to the handling editor at the time of review.

The remaining authors declare that the research was conducted in the absence of any commercial or financial relationships that could be construed as a potential conflict of interest.

## Publisher’s note

All claims expressed in this article are solely those of the authors and do not necessarily represent those of their affiliated organizations, or those of the publisher, the editors and the reviewers. Any product that may be evaluated in this article, or claim that may be made by its manufacturer, is not guaranteed or endorsed by the publisher.
